# A prebiotic, Celmanax™, decreases *Escherichia coli *O157:H7 colonization of bovine cells and feed-associated cytotoxicity in vitro

**DOI:** 10.1186/1756-0500-4-110

**Published:** 2011-04-07

**Authors:** Danica Baines, Stephanie Erb, Ross Lowe, Kelly Turkington, Emil Sabau, Gretchen Kuldau, Jean Juba, Luke Masson, Alberto Mazza, Ray Roberts

**Affiliations:** 1 Lethbridge Research Centre, 5403 1 Avenue South, P.O. Box 3000, Lethbridge, AB, T1J 4B1, Canada; 2 Lacombe Research Centre, 6000 C and E Trail Lacombe, AB, T4L 1W1, Canada; 3 Emil Veterinary Services Ltd, 339 Highway Av, Picture Butte, AB, T0K 1V0, Canada; 4 PENNSTATE, 321 Buckhout Laboratory, University Park, PA, 16802-4508, USA; 5 Fusarium Research Center, PENNSTATE, 216 Buckhout Laboratory, University Park, PA, 16802, USA; 6 Biotechnology Research Institute, National Research Council of Canada, Montréal, QC, H4P 2R2, Canada; 7 Lethbridge Animal Clinic, 3333 1 Avenue South, Lethbridge, AB T1J 4H1, Canada

## Abstract

**Background:**

*Escherichia coli *O157:H7 is the most common serovar of enterohemorrhagic *E. coli *associated with serious human disease outbreaks. Cattle are the main reservoir with *E. coli *O157:H7 inducing hemorrhagic enteritis in persistent shedding beef cattle, however little is known about how this pathogen affects cattle health. Jejunal Hemorrhage Syndrome (JHS) has unclear etiology but the pathology is similar to that described for *E. coli *O157:H7 challenged beef cattle suggestive that *E. coli *O157:H7 could be involved. There are no effective treatments for JHS however new approaches to managing pathogen issues in livestock using prebiotics and probiotics are gaining support. The first objective of the current study was to characterize pathogen colonization in hemorrhaged jejunum of dairy cattle during natural JHS outbreaks. The second objective was to confirm the association of mycotoxigenic fungi in feeds with the development of JHS and also to identify the presence of potential mycotoxins. The third objective was to determine the impact of a prebiotic, Celmanax™, or probiotic,  Dairyman's Choice™ paste, on the cytotoxicity associated with feed extracts *in vitro*. The fourth objective was to determine the impact of a prebiotic or a probiotic on *E. coli *O157:H7 colonization of mucosal explants and a bovine colonic cell line *in vitro*. The final objective was to determine if prebiotic and probiotic feed additives could modify the symptoms that preceded JHS losses and the development of new JHS cases.

**Findings:**

Dairy cattle developed JHS after consuming feed containing several types of mycotoxigenic fungi including *Fusarium culmorum*, *F. poae*, *F. verticillioides*, *F. sporotrichioides*, *Aspergillusflavus*, *Penicillium roqueforti, P. crustosum, P. paneum *and *P. citrinum*. Mixtures of Shiga toxin - producing *Escherichia coli *(STEC) colonized the mucosa in the hemorrhaged tissues of the cattle and no other pathogen was identified. The STECs expressed Stx1 and Stx2, but more significantly, Stxs were also present in the blood clot blocking the jejunum. Mycotoxin analysis of the corn crop confirmed the presence of fumonisin, NIV, ZEAR, DON, 15-ADON, 3-ADON, NEO, DAS, HT-2 and T-2. Feed extracts were toxic to enterocytes and 0.1% Celmanax™ removed the cytotoxicity *in vitro*. There was no effect of Dairyman's Choice™ paste on feed-extract activity *in vitro*. Fumonisin, T-2, ZEAR and DON were toxic to bovine cells and 0.1% Celmanax™ removed the cytotoxicity *in vitro*. Celmanax™ also directly decreased *E. coli *O157:H7 colonization of mucosal explants and a colonic cell line in a dose-dependent manner. There was no effect of Dairyman's Choice™ paste on *E. coli *O157:H7 colonization *in vitro*. The inclusion of the prebiotic and probiotic in the feed was associated with a decline in disease.

**Conclusion:**

The current study confirmed an association between mycotoxigenic fungi in the feed and the development of JHS in cattle. This association was further expanded to include mycotoxins in the feed and mixtures of STECs colonizing the severely hemorrhaged tissues. Future studies should examine the extent of involvement of the different STEC in the infection process. The prebiotic, Celmanax™, acted as an anti-adhesive for STEC colonization and a mycotoxin binder *in vitro*. Future studies should determine the extent of involvement of the prebiotic in altering disease.

## Findings

Enterohemorrhagic *Escherichia coli *O157:H7 continues to cause serious human disease outbreaks despite extensive efforts at cattle processing facilities and feedlots to reduce the transmission of disease [1-3]. Cattle are the main reservoir for *E. coli *O157:H7 and like humans, the majority of cattle resolve experimental-challenge infections on their own, while a few highly colonized individuals develop hemorrhagic enteritis [4]. It is currently unclear why earlier *E. coli *O157:H7 challenge studies did not report pathology, but we hypothesized that an unknown factor such as feed or water quality may be contributing to the development of disease. If *E. coli *O157:H7 does cause disease in cattle, the most likely candidates are diseases with unclear etiologies. Jejunal Hemorrhage Syndrome (JHS) is an emerging hemorrhagic disease in dairy cattle that has been associated with the presence of mycotoxigenic fungi in feed and the presence of *Clostridiumperfringens *type A in hemorrhaged tissues, but there is some question as to whether this pathogen actually plays a role [5]. Current treatments for JHS are ineffective suggesting that a novel approach to disease management must be undertaken. In monogastric livestock, probiotic and prebiotic feed additives mitigate pathogen issues in a number of ways including enhancing the presence of beneficial bacteria in the intestinal tract [6], improving mucosal barrier function [7,8], increasing mucus production [9], inhibiting proinflammatory cytokines [10], enhancing the innate immune response [11], binding pathogen toxins [12] and interfering with colonization sites [8,13]. Very little information is available concerning the benefits of probiotics or prebiotics for managing pathogens in ruminants. Probiotics do reduce *E. coli *O157:H7 shedding in experimental-challenged immature calves [14] and adult cattle [15]. To be effective as pathogen mitigation agents, probiotics or prebiotics must escape fermentation in the rumen and digestion in the abomasum prior to reaching any pathogen colonization sites in the intestinal tract [13]. There is evidence to suggest that prebiotics do survive the rumen environment [16] and as such, these agents may be useful for addressing pathogen issues and disease. The first objective of the current study was to characterize the pathogens associated with the hemorrhaged tissues during natural JHS outbreaks in dairy cattle. The second objective was to confirm the association of JHS development with the presence of mycotoxigenic fungi in feed and to determine if their mycotoxins or cytotoxins were also present. The third objective was to determine the impact of a prebiotic, Celmanax™, and a probiotic, Dairyman's Choice™ paste, on feed extract cytotoxicity *in vitro*. The fourth objective was to determine the impact of the prebiotic and probiotic on *E. coli *O157:H7 colonization of mucosal explants and a bovine colonic cell line *in vitro*. The final objective was to monitor the impact of the prebiotic and probiotic added to alleviate the reduction in milk production on the neurological symptoms and JHS development in dairy cattle during a natural JHS outbreak.

### Dairy production site 1 (PS1) with no history of JHS

A natural outbreak of JHS occurred in a commercial dairy herd in December 2008 and January 2009 in Southern Alberta, Canada. From 1 to 3 animals per week developed disease symptoms and succumbed to the disease despite antibiotic interventions. Eleven mature cattle developed JHS and an average of 10 other animals per week had the neurological symptoms that preceded the development of JHS. The initial diagnosis was Acute Hemorrhagic Enteritis (AHE), however after further examination of tissues, this was changed to JHS. The principle difference in the diagnosis of these diseases is the location and extent of hemorrhaging in the intestinal tract. JHS presents with severe hemorrhaging in the jejunum with a large blood clot blocking the movement of digesta while AHE presents with hemorrhaging in several regions of the intestinal tract with smaller blood clots. The producer reported symptoms suggestive of a mycotoxicosis including lower milk production, staggering, hindlimb paralysis, wasting and higher than average mortality for the lactating cattle. To alleviate the reduction in milk production, the producer added a prebiotic (30 g) to the total mixed ration and a single application of a prebiotic + probiotic (400 ml:10 g) delivered to cattle within 24 hr after calving. The prebiotic, Celmanax™ (Vi-COR^®^, Mason City, IA, USA), consists of a non-living formulation of yeast cell walls or mannan oligosaccharide (MOS) and yeast metabolites. The probiotic, Dairyman's Choice™ paste (Animal Pro-Products, Arthur, ON, Canada), consists of two *Bacillus *isolates that provide enzymes for assisting digestion, vitamins and minerals. The Celmanax™ improves milk production in dairy cattle [17], while the Dairyman's Choice™ paste is applied during times of increased nutritional demands. We monitored the impact of the feed additives on the current neurological symptoms that preceded the development of JHS disease, the occurrence of new neurological symptoms and the development of new JHS cases.

### Dairy production sites 2, 3 and 4 (PS2, PS3, PS4) with no previous history of JHS

From 2008 to 2010, other dairy production sites in Southern Alberta were experiencing sudden-onset cattle deaths, grey-green scours, reduced milk production, metritis, neurological symptoms (staggering), drooling, swollen joints and wasting associated with the consumption of mouldy feeds. Jejunal tissues from one animal per site were sampled. From 6 to 30 mature cattle developed JHS on each site with an average of 100 lactating cattle per site. As for PS1, an average of 1 animal per week had the neurological symptoms that preceded the JHS losses. The producers applied the prebiotic and probiotic to alleviate the reduction in milk production and we monitored the impact of the feed additives on the current neurological symptoms, the occurrence of new neurological symptoms and the development of new JHS cases.

### Dairy production site 5 (PS5) with a history of JHS

A final production site was selected where the veterinarian had annual JHS cases in the herd. No feed additives were provided to the dairy cattle.

### *Escherichia coli *O157:H7 isolation from PS1-PS5

One cow from PS1 died from complications associated with a c-section and the tissue served as a negative control. This animal had no neurological symptoms or other disease symptoms. One animal from PS2 and PS3 died from complications associated with leucosis and reproduction respectively. These animals served as negative controls and did not have any neurological symptoms. The tissue from 11 cattle confirmed the JHS status on PS1 based on the presence of acute hemorrhaging in the jejunum, blood clots in the small intestine and the lack of similar pathology in the control animals. To compare pathology associated with JHS diagnosis at multiple production sites, tissue samples were collected from PS1-PS4 and the pathology recorded. Finally, a sample was obtained from another veterinary practice (PS5) with a production site that had a history of JHS to determine whether there were any obvious differences in the gross pathology compared to PS1 to PS4. For all production sites, a 30 to 60 cm piece of tissue was removed from the acute hemorrhaged region of the jejunum within 4 hours after death and transported back to the laboratory at 4°C. The bloody digesta and clot were separated into sample tubes and serial dilutions were plated on Sorbitol MacConkey agar (SMAC; Dalynn Biologicals, Calgary, Alberta, Canada) to identify non-sorbitol fermenting bacterial colonies and on Potato Dextrose agar (PDA; Dalynn Biologicals, Calgary, Alberta, Canada) to isolate fungi. Adherent or tissue-colonizing bacteria were collected from the hemorrhaged regions. Each tissue was cut open, washed in cold PBS and 2.5 cm^2 ^pieces cut out. Care was taken while cutting not to press down on the tissue and distort size. Each 2.5 cm^2 ^intact tissue was incubated with 0.1% Triton X-100 overnight and plated on SMAC agar to determine the presence of non-sorbitol fermenting bacterial colonies. Any suspect colonies were tested as O157 and H7 using the RIM™ E. coli O157:H7 Latex test (Fisher Scientific, Ottawa, Ontario, Canada). To elucidate whether STEC virulence determinants were critical to the development of JHS, the blood clot that blocked the hemorrhaged jejunum and the *E. coli *O157:H7 isolates from the hemorrhaged regions were examined for Stx1 and Stx2 expression using the Meridian ImmunoCard STAT!^® ^EHEC test (Somagen, Edmonton, Alberta, Canada). The Meridian ImmunoCard STAT!^® ^EHEC test was optimized for use with cattle samples as follows: three-day old colonies on SMAC plates were used in the agar plate method, polymyxin B at 100 μg/ml, a longer Polymyxin B incubation period (45 min) and a 300 μl sample was applied to the test well. For the detection of Stx1 and Stx2 in the blood clot, a 1 ml volume of the blood was diluted in half with PBS and 300 ul was then applied to the test well.

### PCR assay targeting O157 STEC and non-O157 STEC genes

Initial isolates were chosen from PS1-PS5 for additional PCR testing based upon their latex agglutination responses. The PS1, PS3 and PS4 isolates produced strong agglutination for both O157 and H7 in the RIM™ E. coli O157:H7 Latex test. One isolate representing this group was tested for identity. The isolates from the remaining sites produced weak agglutination with O157 and none to strong agglutination with H7 in the RIM™ E. coli O157:H7 Latex test. One isolate representing this group was tested for identity. The presumptive *E. coli *O157:H7 colonies were subjected to an *E. coli *O157:H7-specific PCR assay targeting the *fliC*, *eaeA*, and verotoxin (*vt*; VT1 and VT2) genes [18]. In this assay, *vt*, *eaeA *and *fliC *PCR products are produced for *E. coli *O157:H7 strains while, *vt *PCR products are produced for non-O157 STEC strains.

### DNA microarray assay targeting pathogenic *E. coli *genes

To further characterize the composition of the infections associated with JHS cases, isolates were re-examined in a DNA microarray assay targeting *E. coli *genes. To select isolates, the samples were transferred to SMAC, CT-SMAC and CHROMagar™ O157 and two to five STEC colonies were further characterized based upon differences in morphology and/or Stx production.

### DNA microarrays

The microarray (MaxiVir1.0) used in this study was based on earlier published work [19] and carries 514 oligonucleotides of 70 bases in length targeting 348 virulence or virulence-related genes and 96 antimicrobial resistance or antimicrobial resistance-related genes found in gram-negative bacteria. The microarray, designed to detect a complete set of virulence genes representative of all *E. coli *pathotypes, includes virulence factors such as adhesins, locus of enterocyte effacement, colicins and microcins, toxins, iron acquisition and transport systems, capsular and somatic antigens, hemolysins and hemaglutinins, as well as newly recognized or putative *E. coli *virulence genes. Antimicrobial resistance genes included in the microarray represent different antimicrobial families such as β-lactams, aminoglycosides, tetracycline, phenicols, trimethoprim, sulfonamide and class I integron. The microarray also carries five positive oligonucleotide controls for *E. coli *derived from the sequences of genes encoding tryptophanase (*tnaA*), beta-glucuronidase (*uidA*), lactose permease (*lacY*), beta-galactosidase (*lacZ*), and glutamate decarboxylase (*gad*). Negative controls added to this microarray consist of oligonucleotides derived from the gene sequences for the green fluorescent protein of *Aequorea victoria*, the lactose permease of *Citrobacter freundii*, and the chlorophyll synthase from *Arabidopsisthaliana*.

### *Escherichia coli *DNA labeling

Bacterial DNA was labeled using Bioprime DNA labeling system (Invitrogen Life Technologies, Burlington, ON, Canada). Fifteen μl of the supernatant containing DNA was added to a final volume of 32.5 μl containing 10 μl of a random primer solution, 0.5 μl of high-concentration DNA polymerase (Klenow fragment, 40 U/μl), 5 μl of a deoxyribonucleosidetriphosphate (dNTP) mixture (1.2 mM dATP, 1.2 mM dGTP, 1.2 mM dTTP, and 0.6 mM dCTP in 10 mM Tris [pH 8.0] and 1 mM EDTA), and 2 μl of 1 mMCy5-dCTP. Labeling reactions were performed in the dark at 37°C for 3.5 h and stopped by the addition of 5 μl Na_2_EDTA 0.5 M (pH 8.0). The labeled samples were then purified with a PureLink PCR purification kit (Invitrogen Life Technologies, Carlsbad, CA) according to the manufacturer's protocol. The amount of incorporated fluorescent Cy5 dye was then quantified by scanning the DNA sample with a NanoDrop ND-1000 spectrophotometer from 200 to 700 nm. Data were analyzed using a Web-based percent incorporation calculator http://www.pangloss.com/seidel/Protocols/percent_inc.html.

### Hybridization of labeled DNA

Microarrays were prehybridized at 50°C for 1 hour under a Lifterslip (25 × 60 mm; Erie Scientific Company, Portsmouth, NH, USA) using a SlideBooster hybridization workstation (model SB800; Advalytix, Germany), with 50 μl of prewarmed (37°C) digoxigenin (DIG) Easy Hyb Buffer (Roche Diagnostics, Laval, Quebec, Canada) supplemented with 5% (vol/vol) bovine serum albumin (1 mg/ml; New England Biolabs Inc., Beverly, MA). After pre-hybridization, the lifterslip was removed by dipping the slides in 0.1 × SSC (saline-sodium citrate) and were air-dried. Before hybridization, the samples were dried and resuspended in 15 μl of hybridization buffer (DIG + 0.1 ug/ul ssDNA) and denatured for five minutes at 95°C. One microgram of labeled genomic DNA was hybridized on the MaxiVir1.0 microarray under a lifterslip (18 × 18 mm). The hybridization was carried out overnight at 50°C in a SlideBooster hybridization workstation. After hybridization, lifterslips were removed by dipping the slides in a 0.1 × SSC and 0.1% SDS (sodium dodecyl sulfate) solution. Post-hybridization washes were performed at 37°C: two washes with 0.1 × SSC and 0.1% SDS for ten and five minutes respectively and one last wash in 0.1 × SSC for five minutes. The microarrays were then air-dried.

Microarray slides were scanned at 5 μm resolution with a ScanArray Lite fluorescent microarray analysis system (Perkin-Elmer, Missasauga, Ontario, Canada). Acquisition of fluorescent spots was performed using the ScanArray Express software (Perkin-Elmer, Foster City, CA). Fluorescent spot intensities were quantified with ImaGene software version 8.0 (BioDiscovery, Inc., El Segundo, CA). All the microarrays were normalized using the same method. For each subarray, the mean value for each set of duplicate spotted oligonucleotides was divided by the correction factor taken from the negative controls spots. This value was then divided by the average of the empty spots to create a signal-to-noise ratio. Oligonucleotide spots with a signal-to-noise fluorescence ratio greater than the established threshold (3 in this case), were considered positive. These ratios were then converted into binary data where a value of 0 indicates a negative probe and a value of 1 a positive probe. A threshold of 3 was chosen because it best represented spot quantification. To verify that the results were accurate, we compared the .bmp image of a given sample and the quantified result.

### Isolation of pathogens from hemorrhaged tissue in dairy cattle

Washed tissue samples (2.5 cm^2^) were placed in 0.1% TritonX-100 overnight and the released bacteria stored at -80°C or serial dilutions were direct plated. The digesta was also stored at -80°C or serial dilutions were direct plated. A 1 to 50 μl aliquot of a diluted sample was applied to CHROMagar™ Salmonella, CHROMagar™ E. coli, CHROMagar™ O157, CHROMagar™ Salmonella Plus, and CHROMagar™ Listeria plates (Dalynn Biologicals, Calgary, Alberta). To confirm identity, the presumptive isolates with the exception of the *Listeria *were subjected to a GN-ID A + B biochemical test (Alere™ Canada, Ottawa, Ontario). Presumptive *Salmonella *were also subjected to a Salmonella Latex Agglutination test (Alere™ Canada, Ottawa, Ontario, Canada). Presumptive *Listeria *was identified using *a Listeria *ID test (Alere™ Canada, Ottawa, Ontario). To detect *Clostridium perfringens *type A, samples were examined for characteristic features using a compound Nikon microscope set at 1000 × magnification. Digesta and tissue smears were examined for the presence of large, rectangular bacilli (rod) with or without spores (ovoid, sub-terminal). Sub-cultures on blood agar were examined for rapid spreading growth.

### Isolation and identification of mycotoxigenic fungi

Feed components were collected from PS1-PS5. A 10 g sub-sample of corn silage, barley silage or hay was finely ground and a 5 ml volume added to a PDA plate. The plate was incubated for 1 to 7 days and individual fungal isolates transferred to new PDA plates. *Fusarium *isolates were identified by examination of micro-morphological characters according to Nelson et al. [20] and by PCR amplification and sequencing of a fragment of the *EF1-a *gene and comparing the sequence with the FUSARIUM-ID database [21]. *Penicillium *isolates were identified by microscopic examination of morphology using the guide by Pitt [22]. *Aspergillus *species were identified by microscopic examination of morphology using the guide provided by Klich [23].

### Extraction of cytotoxins from mouldy feed

The methods used have been described previously [24]. The corn silage was almost entirely moldy having a dark burgundy-red coloration for PS1 and visible pockets of white to blue mould were present in the barley silage. The barley silage had visible pockets of white and blue mold for PS2-PS5, while the hay had a pink coloration for PS3 and PS4. To extract the silages and hay, each sample was ground, a 25 ml aliquot of 50% ethanol or methanol was added to a 3 g sample of ground material and placed on a shaker at 200 rpm for 3 h. The supernatant was collected in another tube, and stored at 4°C until use.

### Mycotoxin analysis

The co-occurrence of mouldy feed and a high number of JHS cases suggested that a field survey of local corn crops was warranted for mycotoxin analysis. Cornfields from PS1 and the surrounding region in Lethbridge County were selected to compare mycotoxin profiles. A minimum of 20 intact cobs were collected from each field, the kernels removed, bagged and sent for commercial analysis (Animal Health Laboratory, University of Guelph, ON, Canada). The samples from PS1 were also submitted for analysis to Charm Sciences, Inc. who did the analysis without charge (Lawrence, MA USA).

### *Escherichia coli *O157:H7 strain and culture conditions

*Escherichia coli *O157:H7 E318N is a human isolate (PT14) that was supplied by A. Borezyk, Enteric Reference Laboratory, Ministry of Health, Toronto, Ontario. The strain was maintained at -80°C in 25% glycerol: 75% Luria-Bertoli (LB) broth (Sigma-Aldrich, Oakville, Ontario, Canada) and was grown statically overnight at 37°C in LB broth (Fisher Scientific, Ottawa, Ontario, Canada) when required. The strain was serially diluted to the desired concentration with phosphate-buffered saline (PBS). Bacterial cell counts were determined by plating on SMAC agar and examined for non-sorbitol fermenting colonies that appeared as colorless colonies.

### *In vitro *organ culture (IVOC) *E. coli *O157:H7 adherence assay

Healthy necropsy jejunal samples were obtained from steers using standard methods [4]. Briefly, jejunal tissues (30 cm) were removed within 2 min of release of the intestinal tract from the carcass and each piece was maintained at 4°C for transport back to the laboratory. Upon arrival, the tissue was cut open, washed using PBS at 4°C and 2.5 cm^2 ^pieces were excised. The IVOC adherence assay was conducted as previously described [4] using the *E. coli *O157:H7 E318N strain. This assay has been established as representing *E. coli *O157:H7 colonization *in vivo *and as a useful model system for *E. coli *O157:H7 colonization in cattle. To compare the ability of varying concentrations of the prebiotic, Celmanax™, to interfere with *E. coli *O157:H7 colonization of cattle intestinal tissue, 10^7 ^CFU/ml of *E. coli *O157:H7 was applied to the mucosal surface of tissue pieces to which 0%, 0.01%, 0.1%, 1% and 10% Celmanax™ diluted in DMEM was previously applied. Similarly, 0%, 0.01%, 0.1%, 1% and 10% Dairyman's Choice™ paste was added to cell monolayers and 10^7 ^CFU/ml of *E. coli *O157:H7 was applied to the mucosal surface. The treated mucosal explants were incubated for 4 h under standard culture conditions (37°C, 95% humidity and 5% CO_2_). After incubation, each tissue was washed six times with PBS to remove any unattached bacteria. The tissue was then turned mucosa-side down in 3 ml of PBS supplemented with 1% Triton X-100 (Sigma-Aldrich, Oakville, Ontario, Canada) and incubated at 4°C overnight. After 24 hr, serial dilutions of the released bacteria were plated on SMAC agar and the number of non-sorbitol fermenting colorless colonies recorded. The Triton X-100 effectively removed all the attached bacteria after 24 hr incubation at 4°C. The assay was replicated three times using the jejunum from three different animals.

### *In vitro *cell culture (IVCC) *E. coli *O157:H7 E318N adherence assay

The methodology was described previously for comparing the colonization potential of *E. coli *O157:H7 strains belonging to different lineages [25]. Briefly, the cell line was cultured in DMEM supplemented with 10% fetal bovine serum (Fisher Scientific, Oakville, Ontario, Canada) and gentamicin (Sigma-Aldrich, Oakville, Ontario Canada) under standard culture conditions. Cells were sub-cultured by trypsinization of the cell monolayer with 0.25% trypsin-EDTA solution (Sigma-Aldrich, Oakville, Ontario, Canada) for 5 min at 37°C, and the supernatant centrifuged at 2 087 × *g *for 10 min. The cell pellet was re-suspended in the culture medium and then, added to each well of a 6-well multi-well Falcon plate (Fisher Scientific, Oakville, Ontario, Canada) at a density of approximately 10^3 ^cells. The cells were grown until they formed a confluent monolayer consisting of 10^5 ^cells.

To determine the impact of the feed additives on *E. coli *O157:H7 E318N colonization of the colonic cell line, each well of confluent cells was washed once with PBS and then, 3 ml of DMEM with varying concentrations of the prebiotic, Celmanax™, or probiotic, Dairyman's choice™ paste, including 0%, 0.01%, 0.05% and 0.1%, was added. The concentrations of Celmanax™ used reflected the percentage achievable under standard feeding regiments for the prebiotics and probiotics given the dilution factor of the rumen and the passage rate for materials in the gastrointestinal tract. For the IVCC bioassay, we did not exceed 0.1% prebiotic or probiotic as it shifted the pH of the media and affected the health of the cells. *Escherichia coli *O157:H7 E318N was added to the cell monolayer to deliver a final exposure dose of 10^6 ^CFU/10^5 ^cells. The cell cultures were incubated for 3 h under standard culture conditions. At the end of the experiment, cell-monolayers were washed with PBS to remove any unattached bacteria and 2 ml of PBS supplemented with 0.1% Triton X-100 was added and incubated overnight at 4°C. Serial dilutions of the released bacteria were performed and quantified by plating onto SMAC plates. Plates were incubated at 37°C overnight and *E. coli *O157:H7 was quantified by counting the non-sorbitol fermenting colonies that appear colorless. The IVCC assay was replicated 6 times with a minimum of 3 different culture dates for the cell line.

### Lawn assay for cytotoxicity associated with mouldy feed extracts and pure mycotoxins

The lawn assay has been described previously [25] and was used to examine the cytotoxicity of feed extracts and mycotoxins in the absence and presence of 0.1% Celmanax™ and Dairyman's Choice™ Paste. For the silage extracts, enterocytes isolated from the jejunum served as the cell lawn and the silage extracts were applied to assess for cytotoxicity. Briefly, a 1% SeaKem^® ^Agarose (Mandel Scientific, Guelph, Ontario, Canada) support gel was poured into a petri dish. Next, the lawn agarose [3 ml of 3.7% SeaPlaque^® ^agarose (Mandel Scientific, Guelph, ON, Canada)] was mixed with 3 ml of cell suspension and poured over the support agarose. For the pure mycotoxins, cell monolayers were prepared as described for the IVCC bioassay. A 400 μl aliquot of 0.4% SeaPlaque^® ^agarose was poured over the cell monolayer and cooled for 15 min. The mycotoxins tested included DON (100 μg/ml), ZEAR (100 μg/ml), T-2 (100 μg/ml), and fumonisin B1 (50 μg/ml; Romer Labs, Union, MO, USA). A 5 μl aliquot of the stock mycotoxins was applied to the center of each cell monolayer. A 5 ul aliquot of the solvent used for the extraction process and the mycotoxin buffer served as negative controls. Each extract or mycotoxin (5 μl) was applied with or without 0.1% prebiotic or probiotic and the plate incubated for 4 h under standard culture conditions. The lawn was stained with 0.1% trypan blue (Sigma-Aldrich) and de-stained using PBS. Plates were scored the same day and the amount of extract cytotoxicity was scored as low (1), moderate (2) or high (3) which was visualized as a faint blue spot, a blue spot or a dark blue spot respectively. These activities were compared to two standards, ground corn containing 0.1 ppm aflatoxin that had a cytotoxicity score of 1 and 1 ppm aflatoxin that had a cytotoxicity score of 3. The assay was repeated a minimum of three times.

### Data analysis

The IVOC adherence assay and IVCC adherence assay data were log transformed to normalize the data prior to analysis. All data were analyzed using ANOVA followed by a posthoc Tukey's test for comparison of the means. For the IVOC and IVCC data, the model consisted of two factors, passage number of the cell line or animal and dose. Results were considered significant if *P *< 0.05 and non-significant if *P *> 0.05.

## Results

### Impact of a prebiotic and probiotic on the symptoms and development of JHS for PS1

The inclusion of Celmanax™ in the total mixed ration was associated with both a cessation of current neurological symptoms and no further development of neurological symptoms in the lactating cattle. The inclusion of Celmanax™ and Dairyman's Choice™ paste for freshening cattle changed the pattern for JHS development from 1 to 3 animals per week to 0 animals per week. This pattern was maintained throughout the winter while the cattle consumed the remaining mouldy corn and barley silage.

### Impact of a prebiotic and probiotic on the symptoms and development of JHS for PS2-4

The inclusion of Celmanax™ and Dairyman's Choice™ in the total mixed ration was associated with both a cessation of the current neurological symptoms and no further development of neurological symptoms in the lactating cattle. The inclusion of Celmanax™ and Dairyman's Choice™paste for freshening cattle changed the pattern for JHS development from 1 animal per week to 0 animals per week. This pattern was maintained throughout the winter while the cattle consumed the remaining mouldy barley silage and/or hay.

### Pathology associated with the jejunum PS1-PS5

Each tissue was examined and the pathology recorded. The control tissues had no pathology present. All hemorrhaged jejunum samples from JHS cases had the following common pathology: inflamed and raised Peyer's Patches in the jejunum, severe jejunal hemorrhages visible through the serosa, bloody digesta visible through the serosa, complete loss of mucosal structure in the hemorrhaged regions, dark-red erythema, large blood clots and edema. The main difference between PS5 and PS1-PS4 was the presence of a much larger blood clot that completely blocked the passage of digesta in the jejunum.

### Shiga toxin - producing *E. coli *in the jejunum of cattle from PS1-PS4 with no history of JHS

Adherent non-sorbitol fermenting colonies were identified at significant levels colonizing the hemorrhaged regions of the jejunum relative to the control animals (*P *= 0.001, Table 1). There were significantly higher amounts of non-sorbitol fermenting colonies identified in the digesta of the jejunum relative to control animals (*P *= 0.001, Table 1). There were also sorbitol-fermenting colonies that represented about 50% of the CFU/sample/site but we did not initially suspect them as non-O157 STECs. The bacterial isolates were confirmed as O157 and H7 using the RIM™ E. coli O157:H7 Latex test, however there was variation in the strength of the agglutination particularly for O157. All isolates were examined for the expression of Stx, as this is a key virulence trait associated with more severe forms of animal disease. All isolates produced Stx1 and Stx2. The consistent presence of significant numbers of STEC in the hemorrhaged regions for cattle with JHS regardless of the production site, suggests that the STEC warrant further investigation as causative agents.

**Table 1:** Average number of STEC CFU per tissue type for dairy cattle with or without JHS.

Sample type	Cattle with JHS (n = 12) Mean Log CFU + SE	Cattle without JHS (n = 3) Mean Log CFU + SE
**Jejunum (per 2.5 cm^2 ^mucosa)^1^**	5.303 ± 0.184a	0.000 + 0.000b

**Digesta (per ml)**	4.487 ± 0.172a	0.000 + 0.000b

1 row numbers followed by different letters are significantly different *P *= 0.001.

### *Escherichia coli *O157:H7 and Stx in the jejunum of a cow from PS5 with a history of JHS cases

Adherent non-sorbitol fermenting colonies were identified at significant levels in the hemorrhaged regions of the jejunum. This animal had similar amounts of *E. coli *O157:H7 present as observed for the other production sites: 7 × 10^5 ^CFU/2.5 cm^2 ^hemorrhaged jejunum, 1.6 × 10^4 ^CFU/ml bloody digesta, 6.0 × 10^4 ^CFU/2.5 cm^2 ^intact severe focal hemorrhage, 1.7 × 10^5 ^CFU/2 cm^2 ^jejunal Peyer's patch, 2.3 × 10^6 ^CFU/2.5 cm^2 ^inflamed jejunum. To confirm a potential role for Stx in the development of JHS pathology, we evaluated the isolate for Stx expression and the blood clot blocking the hemorrhaged jejunum for the presence of Stx. The isolate did express Stx1 and Stx2, but more significantly, both toxins were detected in the blood clot.

### Isolation of pathogens from dairy cattle with JHS

Bacterial pathogens in the digesta and hemorrhaged mucosa were isolated from the jejunum. CHROMagar™ is a selective media used for presumptive identification of specific pathogens including *Salmonella*, *E. coli*, *E. coli *O157 and *Listeria*[26-28]. Presumptive *E. coli *O157 were identified and appeared as regular mauve colonies (O157 STEC and non-O157 STEC), irregular mauve colonies or navy blue colonies with a small to large mauve halo (non-O157 STEC) on CHROMagar™ O157 plates. Presumptive *E. coli *O157 were confirmed as pathogenic *E. coli *in the GNID A + B test. Other presumptive pathogenic *E. coli *appeared as blue colonies on CHROMagar™ *E. coli *plates. All *E. coli *were confirmed as non-pathogenic *E. coli *in the GNID A + B test. White colonies were also found on this medium that were mauve on CHROMagar™ *E. coli *O157 and were identified as pathogenic *E. coli *in the GNID A + B Test. This provided a crosscheck for the results from the CHROMagar™ *E. coli *O157 and for the non-sorbitol or sorbitol fermenting colonies. Presumptive *Salmonella *were identified and appeared as mauve colonies on CHROMagar™ Salmonella plus plates, but were subsequently determined as false positives (pathogenic *E. coli *using the GNID A+ B kit and the Salmonella Latex Agglutination kit). Presumptive *Listeria *species were identified and appeared as pinpoint blue colonies with a halo on CHROMagar™ *Listeria *plates and these isolates were later determined to be *Listeria grayi*, a non-pathogenic strain. No *Clostridiumperfringens *type A was detected. Thus, the only pathogen identified in the hemorrhaged tissues or bloody digesta for cattle with JHS regardless of the production site, were STECs.

### PCR assay targeting O157 STEC and non-O157 STEC

The variation in the strength of the latex agglutination test and the possible identification of potential sorbitol-fermenting STEC suggested that closely related STECs were present in the tissues rather than *E. coli *O157:H7 alone. To confirm that not all isolates were *E. coli *O157:H7, PCR products produced from each isolate were characterized. The *vt*, *eaeA *and *fliC *PCR products were detected for isolates giving strong latex agglutination for O157 and H7 in the RIM™ E. coli O157:H7 Latex test defining the isolates from PS1, PS3 and PS5 as *E. coli *O157:H7. In contrast, only *vt *PCR products were detected for isolates giving weak latex agglutination for O157 and H7 in the RIM™ E. coli O157:H7 Latex test defining the isolates from PS2 and PS4 as non-O157 STECs.

### DNA microarray assay targeting *E. coli *genes

The results from the bacterial isolations from the SMAC, CT-SMAC and CHROMagar™ O157 plates suggested that the tissues had multiple types of STEC present. There were several distinct morphologies on the CHROMagar™ O157 plates: mauve colonies with a circular form, raised elevation and entire or undulate margin; blue colonies with a mauve halo that had a circular form, raised elevation and an entire or undulate margin. These colonies were sorbitol or non-sorbitol-fermenting and tellurite-resistant. Two common STEC morphologies were observed in all tissues: regular mauve colonies having a circular form, raised elevation and entire margin; blue colonies with a mauve halo having a circular form, raised elevation and entire margin. The DNA microarray assay confirmed that mixtures of non-O157 STEC were colonizing the hemorrhaged tissues and these isolates have the locus of enterocyte effacement (LEE) pathogenicity island, *stx1A*, *stx1B *and *hlyA*. Finally, in addition to the genes mentioned above, variability in the presence of other virulence genes also supports the notion of a mixed STEC population. Interestingly, the absence of the *stx2 *gene in all the characterized isolates suggests that the detected Stx2 from the isolate and blood samples may represent a strain that was not ultimately isolated.

### Mycotoxigenic fungi in silages for the production sites

Unlike previous reports for JHS, there was no *A. fumigatus *present in the silage samples from PS1 (Table 2). There were several types of mycotoxigenic fungi present in the corn silage including *F. culmorum*, *A. flavus*, *P. roqueforti, P. crustosum, P. paneum and P. citrinum*. There were several fungi present in the barley silage including *F. poae*, *F. verticillioides*, *F. sporotrichioides*, *P. roqueforti*, *P. citrinum *and *A. flavus *(Table 2). We also identified several fungi in the hemorrhaged regions from the dairy cattle with JHS including *P. roqueforti, Trichothecium roseum, A. flavus and P. citrinum*. For PS2-PS4, there was no *A. fumigatus *present in the barley silage or hay. There were several fungi present in the barley silage including *F. poae*, *F. verticillioides*, *F. sporotrichioides*, *P. roqueforti*, *P. citrinum *and *A. flavus (*Table 2). There were fewer fungi present in the hay including *F. poae, P. roqueforti, P. crustosum *and *P. paneum*. The *Fusarium *species were present in the hemorrhaged regions from the dairy cattle with JHS.

**Table 2:** Percent of samples positive for mycotoxigenic fungi for each corn silage, barley silage and hay sample from production sites (n = 5-10).

Mycotoxigenic Fungi	Corn Silage	Barley Silage	Hay
*Fusarium culmorum*	100	0	0

*F. poae*	0	100	100

*F. verticillioides*	0	100	0

*F. sporotrichioides*	0	100	0

*Aspergillusflavus*	100	20	0

*Penicillium roqueforti*	100	100	100

*P. crustosum*	20	0	20

*P. paneum*	20	0	20

*P. citrinum*	50	25	0

### Mycotoxin Analysis

There were no mycotoxins detected for the corn silage in PS1 using the ROSA^® ^Fumonisin test, ROSA^® ^Aflatoxin test, ROSA^® ^Zear test or ROSA^® ^Don test. The samples were categorized as very dirty which could result in matrix interference or be difficult to analyze using standard commercial methods. There were several mycotoxins detected using commercial HPLC methods for the corn from eight fields in Lethbridge County including PS1 (Table 3). Because the bloody gut symptoms suggested that fumonisin or T-2 toxin could be involved, an additional analysis for fumonisin was performed using LC/MS/MS. The fumonisin B1 levels were very high for PS1 and one other field that was part of a commercial business providing corn to producers for silage production.

**Table 3:** Quantitification of mycotoxins in corn from eight production sites in Lethbridge County.

Mycotoxin	ppm
DON	0.06-0.30

15-DON	0.05-0.08

3-ADON	0.05

NEO	0.07

DAS	0.06

HT-2	0.04

T-2	0.06

NIV	0.12

Aflatoxin B1	0.001

Aflatoxin B2	0.0003

Aflatoxin G1	0.002

Aflatoxin G2	0.0005

ZEAR	0.04

Ochratoxin A	0.004-1.5

Fumonisin B1	26-43

Fumonisin B2	5.1-9.4

### IVOC *E. coli *O157:H7 adherence assay

**Figure 1:**
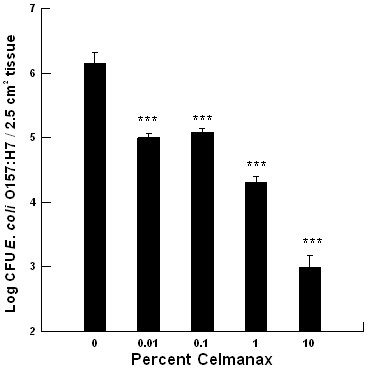
**The impact of varying concentrations of Celmanax™ on *Escherichia coli *O157:H7 E318N colonization of mucosal explants from the jejunum (*n *= 3)**. The 2.5 cm^2 ^mucosal explant was exposed to 10^7 ^CFU (***, *P *= 0.001).

The Dairyman's Choice™ paste had no effect on *E. coli *O157:H7 colonization of the mucosal explants *in vitro *(data not shown). There was a significant dose-dependent reduction in *E. coli *O157:H7 colonization of the mucosal explants from the jejunum in response to varying concentrations of Celmanax™ (*P *= 0.001, Figure 1). The threshold dose for the reduction in *E. coli *O157:H7 colonization of the mucosal explants was 0.01% Celmanax™ when compared with the control (*P *= 0.001, Figure 1). This resulted in about a 10-fold reduction in colonization per tissue. Thereafter, there was a continued decline in *E.coli *O157:H7 colonization with a maximum effect at 1% Celmanax™ when compared with the control (*P *= 0.001, Figure 1). This resulted in about a 100-fold reduction in colonization per tissue.

### IVCC *E. coli *O157:H7 adherence assay

**Figure 2:**
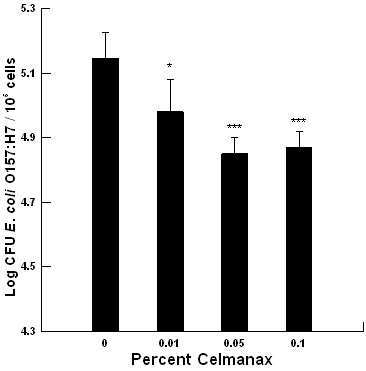
**The impact of varying concentrations of Celmanax™ on *Escherichia coli *O157:H7 E318N colonization of a colonic cell line (*n *= 6-8)**. A confluent monolayer of a bovine colonic cell line (10^5 ^cells) was exposed to 10^5 ^CFU (*, *P *= 0.05; ***, *P *= 0.001).

The Dairyman's Choice™ paste had no effect on *E. coli *O157:H7 colonization of the bovine colonic cell line *in vitro *(data not shown). There was a significant dose-dependent reduction in *E. coli *O157:H7 colonization of the bovine colonic cell line in response to varying concentrations of Celmanax™ (*P *= 0.001, Figure 2). There was a significant passage effect (p = 0.001) with no passage/treatment interaction suggesting that Celmanax™ affected pathogen colonization regardless of passage number. The threshold dose for the reduction in *E. coli *O157:H7 colonization of the colonic cell line was 0.01% Celmanax™ when compared with the control (*P *= 0.05, Figure 2). This resulted in about a 1.6-fold reduction in colonization per 10^5^cells. Thereafter, there was a continued decline in *E. coli *O157:H7 colonization with a maximum effect at 0.1% Celmanax™ when compared to the control (*P *= 0.001, Figure 2). This resulted in a 2.5-fold reduction in colonization per 10^5 ^cells. Higher doses could not be evaluated as they shifted the pH to favor acidic conditions. Overall, the colonic cell line provided similar *E. coli *O157:H7 colonization responses to the inclusion of the prebiotic, but at a lower sensitivity when compared with the IVOC bioassay.

### Lawn assay for cytotoxicity associated with feed extracts and mycotoxins

**Figure 3:**
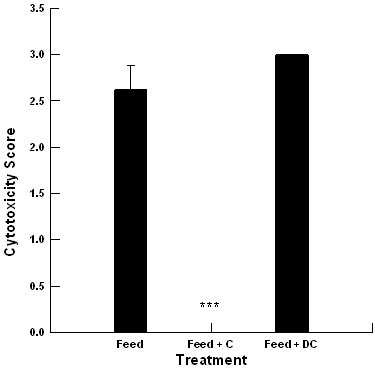
Impact of 0.1% Celmanax™ (C) and Dairyman's Choice™ (DC) paste on the cytotoxicity of methanol extractions from feed (n = 24 for feed extracts, n = 24 for Feed + C, n = 6 for Feed + DC; ***, *P = 0.001*).

The 50% methanol and 50% ethanol extracts from feed components were equally cytotoxic to enterocytes. At least one or more of the mouldy feed components had similar cytotoxicity scores regardless of production site (*P *> 0.05), as such the data was pooled and it is presented in Figure 3.

The extracts from the corn silage from PS1 produced cytotoxicity equivalent to a 1 ppm aflatoxin (in corn) standard or cytotoxicity score of 3. Equivalent extracts from the barley silage produced a ten fold lower cytotoxicity equivalent to a 0.1 ppm aflatoxin (in corn) standard or a cytotoxicity score of 1. A 0.1% concentration of Celmanax™ eliminated the silage extract cytotoxicity regardless of the extraction buffer or the silage type. A 0.1% concentration of Dairyman's Choice™ paste had no effect on silage extract cytotoxicity (Figure 3, *P *> 0.05). The 50% methanol extracts of the barley silages from PS2-PS5 were equally cytotoxic to enterocytes producing cytotoxicity equivalent to 1 ppm aflatoxin (in corn) standard or a cytotoxicity score of 3. However, PS3 and PS4 also had the same activity associated with the 50% methanol hay extracts. Similarly, a 0.1% concentration of Celmanax™ significantly reduced the extract cytotoxicity regardless of the extraction buffer or the feed type *(P *= 0.001).

The DON, T-2, ZEAR and fumonisin B1 were cytotoxic to a colonic cell line from cattle (Table 4). The cytotoxicity score was equivalent to a 1 ppm aflatoxin (in corn) standard or an average cytotoxicity score of 2 or 3 depending upon the mycotoxin. A 0.1% concentration of Celmanax™ significantly reduced the cytotoxicity regardless of mycotoxin type (*P *= 0.001, Table 4). The cytotoxicity of all feed extracts and mycotoxins were essentially eliminated by the presence of Celmanax™ suggesting that this prebiotic is able to interfere with the ability of the mycotoxin to interact with the target cells.

**Table 4:** Impact of 0.1% Celmanax™ and Dairyman’s Choice™ paste on the  cytotoxicity of T-2, DON, ZEAR and Fumonisin B1, n=3.

Treatment	Cytotoxicity Index ^1,2 ^(Mean ± S.E.)
Control (50% methanol)	0 ± 0 a

DON	3 ± 0 b

ZEAR	2 ± 0.6 b

T-2	3 ± 0 b

FUM B1	3 ± 0 b

Celmanax™ + DON	0 ± 0 a

Celmanax™ + Zear	0 ± 0 a

Celmanax™ + T-2	0 ± 0 a

Celmanax™ + FUM B1	0 ± 0 a

Dairyman's Choice™ + DON	3 ± 0 b

Dairyman's Choice™ + ZEAR	2 ± 0.6 b

Dairyman's Choice™ + T-2	3 ± 0 b

Dairyman's Choice™ + FUM B1	3 ± 0 b

^1 ^0 = no cytotoxicity or no blue spot; 1 = about 25% cell death or faint blue spot; 2 = about 50% cell death or blue spot; 3 = about 100% cell death or dark blue spot.

^2 ^column means followed by different letters are significantly different from each other *P *< 0.001.

## Discussion

JHS is a hemorrhagic disease in dairy cattle with the main pathology presenting as hemorrhaging, severe inflammation, mucosal erosion and a major blood clot blocking the jejunum [5]. Two factors are being investigated as causative agents namely mouldy feeds containing *A. fumigatus *and a pathogen *C. perfringens *type A. In the current study, we confirmed the presence of mycotoxigenic fungi but not *A. fumigatus *in the mouldy feeds consumed by cattle that developed JHS. In addition, we further expanded this association to include mycotoxins in feed components. Specific mycotoxins such as T-2 toxin do promote gastrointestinal lesions [29], but they do not induce hemorrhaging on their own. Since fumonisin B1 was present at significant levels in the corn crop and it can cause abdominal pain or diarrhea upon ingestion [30], the pain and abnormal feces observed in the current study suggest that there may be an interaction between pathogen colonization, mycotoxin concentrations and the development of JHS. In earlier JHS studies targeting the identification of potential pathogens, *C. perfringens *type A was detected, but there was some question as to whether this was relevant as it is a normal inhabitant of the intestine that quickly overgrows upon death. In the current study, no *C. perfringens *type A was detected but significant O157 STEC and non-O157 STEC were colonizing the hemorrhaged mucosa. STECs do cause diarrhea in other animal model systems [31] and their presence in the hemorrhaged region merits further investigation. With the exception of experimental challenge studies with *E. coli *O157:H7 [4], these pathogens are not known to cause disease in mature cattle. However, it is possible that this class of pathogens has been overlooked as most of the animals succumbing to JHS do so at production sites. If STECs are pathogens of cattle, they should express similar virulence traits described for serious forms of disease in other animals. One virulence determinant, Stx expression, is critical for the development of severe disease symptoms in susceptible animals [32-34]. Stxs have both lethal [35-37] and sublethal [38] effects on cells that benefit pathogen colonization by providing a foothold in the mucosa for replication and increasing the availability of non-intimate attachment sites. All of the STECs isolated from the cattle diagnosed with JHS expressed Stx and the Stxs were present in the blood clot that blocked the jejunum suggesting that the STECs did have the appropriate virulence traits to elicit serious disease. Future research should focus on examining the contribution of mycotoxins and STECs to the infection process associated with JHS.

Cattle supplemented with prebiotics or probiotics show improvements in performance measurements such as milk production and feed conversion efficiencies [15,39-42]. Improvements in animal health are limited to observations of lower incidences of scours in calves [43], improved immune function in calves [44] or lower somatic cell counts in cattle [39,45]. Given that both types of products can alter rumen microbial populations [16,40] and prevent rumen acidosis [41,42], these benefits have always been interpreted as an improvement in rumen function. Only a few animal studies have examined the potential use of probiotics or prebiotics to inhibit enteric pathogens such as *E. coli *O157:H7 in the gastrointestinal tract of cattle [14,15] and as far as we are aware, no cattle tissue or cell studies have been attempted. In monogastric livestock, prebiotics directly act as anti-adhesives against *E. coli *O157:H7 by mimicking host cell receptor sites [46] and mucin binding sites [8]. This reduces both the amount of *E. coli *O157:H7 colonization and the number of bacteria/microcolony. In the current study, the absence of an active immune system in the IVOC or IVCC bioassay together with the decrease in *E. coli *O157:H7 colonization in the presence of Celmanax™ supports an anti-adhesive behavior for the prebiotic. The second benefit of the prebiotic was that it blocked the cytotoxicity associated with the silages and the pure mycotoxins *in vitro *suggesting that it binds-up the toxic elements in a way that prevents cellular interactions. In contrast, the Dairyman's Choice™ paste had no such effect in the bioassays. The administration of the prebiotic or the prebiotic + probiotic to the cattle was associated with a cessation of neurological symptoms, no further development of neurological symptoms and no further development of new JHS cases. Future studies should examine the mechanisms responsible for the prebiotic or prebiotic:probiotic associated reduction in disease.

Prebiotics are non-digestible mannan oligosaccharides (MOS) that are used in feed rations of cattle to improve performance parameters [2]. These products are obtained from disrupting cell walls of yeast with each product claiming to have a more effective, ruminant-specific starter strain or formulation. Other ingredients can also be present in prebiotic formulations including d-mannose which blocks pathogen type-1 pili binding to receptor sites [6,45], glucomannan which absorbs feed-related toxins [12,42,46], and a yeast extract that provides a concentrated B vitamin supplement that can induce innate immune responses [10,11]. The impact of prebiotics on livestock performance is variable [44,47,48] suggesting that the type of oligosaccharide or added ingredients may influence the measured changes in animal performance. In cattle, to be effective, prebiotics must navigate through the fermentation/digestion processes of the upper gastrointestinal tract prior to interacting with target sites in the intestinal tract. Therefore, the variation in responsiveness reported in studies could be related to the persistence of the prebiotic or additional ingredients in the gastrointestinal tract. The lower incidence of disease associated with prebiotic use in cattle [44,45] together with the results of the current study suggest that a prebiotic, such as Celmanax™, warrant further investigation as disease management tools.

*Escherichia coli *pathogens initially adhere to the mucus layer in the intestinal tract prior to colonization of target intestinal cells [49]. The IVOC or mucosal explant bioassay has a mucus layer and target cells while the IVCC bioassay has cells only. This suggests that the observed differences in bioassay sensitivity are related to the impact of the mucus layer on pathogen colonization. In addition, the larger reduction associated with the mucosal explant bioassay suggests a greater impact than predicted with the cell line bioassay *in vivo*. Previous studies with monogastric cell lines have shown that 1.6% prebiotic (GOS) reduced up to 70% *E. coli *pathogen colonization of Caco-2 and Hep-2 cells, while 0.1% prebiotic reduced *E. coli *pathogen colonization of Caco-2 cells by 10% [9]. In contrast, 1% Celmanax^™ ^reduced up to 98% O157 STEC colonization of mucosal explants and 0.1% Celmanax™ reduced up to 50% *E. coli *O157:H7 colonization of the bovine colonic cell line *in vitro*. Together, these results suggest that prebiotics could significantly alter STEC colonization at the mucosa and the amount of anti-adhesive behavior may be related to chemical composition. Future studies should characterize the mode of action of prebiotics to provide the basis for evaluating them as pathogen mitigation agents.

## Conclusion

The current study confirmed that cattle developed JHS disease after consuming feed containing mycotoxigenic fungi, but also expands this association to include the mycotoxins deposited in the feed. Unlike earlier studies, we did not confirm the presence of *C. perfringens *type A. A mixture of novel pathogens was colonizing the hemorrhaged tissues, O157 STEC and non-O157 STEC. Shiga toxins were expressed by the STECs and were present in the blood clot blocking the hemorrhaged jejunum. A prebiotic, Celmanax™, acted as an anti-adhesive agent for STEC colonization and a mycotoxin binder *in vitro*. The addition of the prebiotic to alleviate milk production losses also was associated with a reduction in disease. Future studies should examine the extent of the involvement of the different STECs in the infection process that is associated with the development of JHS. In addition, further characterization of the mode of action of prebiotics is required to adequately assess them as disease management tools for cattle.

## Competing interests

The authors declare that they have no competing interests.

## Authors' contributions

DB conceived of the study, designed the study, collected tissue, performed the mucosal studies, plated fungi, extracted silages, performed the lawn assay, performed the statistical analysis and drafted the manuscript; RL performed the IVCC bioassay; SE prepared the cells for the IVCC bioassay, plated fungi, extracted silages, performed lawn assays; ES provided JHS tissue samples; KT provided morphological identification of the fungi; GK provided morphological and PCR identification of fungi; JJ preformed the PCR bioassays for Fungal identification; LM conceived of the study to characterize the O157 STEC and non-O157 STEC; AM performed the DNA microarrays for STEC identification; RR provided tissues samples, consulted on pathology and disease diagnosis. All authors have read and approved the manuscript.
